# TOP2A modulates signaling via the AKT/mTOR pathway to promote ovarian cancer cell proliferation

**DOI:** 10.1080/15384047.2024.2325126

**Published:** 2024-03-06

**Authors:** Kaiwen Zhang, Xingyu Zheng, Yiqing Sun, Xinyu Feng, Xirong Wu, Wenlu Liu, Chao Gao, Ye Yan, Wenyan Tian, Yingmei Wang

**Affiliations:** a Department of Gynecology and Obstetrics, Tianjin Medical University General Hospital, Tianjin, China; b Department of Gynecology and Obstetrics, Affiliated Hospital of Nantong University, Nantong, China

**Keywords:** Ovarian cancer, TOP2A, prognosis, proliferation, rescue experiments, AKT/mTOR signaling pathway

## Abstract

Ovarian cancer (OC) is a form of gynecological malignancy that is associated with worse patient outcomes than any other cancer of the female reproductive tract. Topoisomerase II α (TOP2A) is commonly regarded as an oncogene that is associated with malignant disease progression in a variety of cancers, its mechanistic functions in OC have yet to be firmly established. We explored the role of TOP2A in OC through online databases, clinical samples, in vitro and in vivo experiments. And initial analyses of public databases revealed high OC-related TOP2A expression in patient samples that was related to poorer prognosis. This was confirmed by clinical samples in which TOP2A expression was elevated in OC relative to healthy tissue. Kaplan-Meier analyses further suggested that higher TOP2A expression levels were correlated with worse prognosis in OC patients. In vitro, TOP2A knockdown resulted in the inhibition of OC cell proliferation, with cells entering G1 phase arrest and undergoing consequent apoptotic death. In rescue assays, TOP2A was confirmed to regulate cell proliferation and cell cycle through AKT/mTOR pathway activity. Mouse model experiments further affirmed the key role that TOP2A plays as a driver of OC cell proliferation. These data provide strong evidence supporting TOP2A as an oncogenic mediator and prognostic biomarker related to OC progression and poor outcomes. At the mechanistic level, TOP2A can control tumor cell growth via AKT/mTOR pathway modulation. These preliminary results provide a foundation for future research seeking to explore the utility of TOP2A inhibitor-based combination treatment regimens in platinum-resistant recurrent OC patients.

## Introduction

Ovarian cancer (OC) is the deadliest form of gynecological malignancy, posing a significant threat to the health of women throughout the globe. In 2020 alone, there were an estimated 313,959 OC diagnoses and 207,252 deaths among affected patients.^1^ In the USA alone, the American Cancer Society predicts that there will be 19,710 cases of OC diagnosed in the USA in 2023, with 13,270 deaths.^2^ While OC is not the most commonly diagnosed cancer of the female reproductive tract, its incidence continues to rise annually. Pathological subtypes of OC include epithelial OC, which is dominant and accounts for > 90% of cases,^3^ while high-grade serous OC is the most frequently diagnosed histological subtype, comprising approximately 75% of all epithelial OC cases.^4^

In its early stages, the symptoms of OC are relatively nonspecific such that an estimated 75% of patients are only diagnosed when the disease is relatively advanced, with a 5-year survival rate of just 29–48%. In patients with platinum-resistant recurrent disease, the survival duration is only 12–14 months on average.^4,5^ As such, there remains a pressing need to design new strategies capable of improving survival outcomes in platinum-resistant patients with recurrent disease.

In patients with platinum-resistant disease, non-platinum drugs are the primary therapeutic approach, including weekly treatment with paclitaxel, docetaxel, the topoisomerase (TOP) IIα inhibitor etoposide, TOP IIβ inhibitor liposomal doxorubicin, and TOP I inhibitor topotecan. However, the therapeutic performance of chemotherapy remains inadequate in these cases, with a response rate of just 20–28% in platinum-resistant patients undergoing monotherapy, and these treatments also entail a range of debilitating side effects,^6–9^ emphasizing the need for combination regimens. Previous clinical trials have confirmed that the combination of apatinib with oral etoposide shows promising efficacy and manageable toxicities in patients with platinum-resistant or platinum-refractory ovarian cancer.^10,11^ As such, ongoing efforts to explore TOP inhibitor-based combination therapy may help to improve patient outcomes.

The DNA TOP enzyme is responsible for regulating DNA topological characteristics during transcription, catalyzing specific topological reactions by cutting and ligating DNA ends. TOP enzymes include type I and type II subtypes,^12^ with type II topoisomerases (TOP2) serving as conserved regulators of chromatin topology that catalyze the reversible formation of double-stranded breaks in the DNA necessary for the maintenance of genomic integrity in the contexts of transcription, replication, and cell division. Topoisomerase IIα (TOP2A) primarily localizes to the nuclear and mitochondrial compartments, regulating double-stranded break formation, binding, and DNA topology to shape transcription, replication, and related processes.^13,14^ TOP2A is also closely associated with the cell cycle, with peak activity in the G2/M phase and reduced levels after mitosis is complete. TOP2A is an essential regulator of mitotic chromosome concentration and separation.^15,16^ In prior reports, TOP2A was found to be closely related to tumor malignancy. In one study, for example, TOP2A protein levels in colon cancer were reported to be increased compared to those in healthy tissues, and TOP2A knockdown could inhibit the proliferation and invasion of colon cancer cells.^17^ Dong et al. also determined that TOP2A is capable of inhibiting liver cancer cell proliferative and invasive activity through the regulation of epithelial-mesenchymal transition.^18^ TOP2A can also shape the progression of metastatic pancreatic neuroendocrine,^19^ prostate,^20^ non-small cell lung,^21^ and cervical tumors.^22^ In OC, TOP2A can reportedly serve as a marker that predicts pegylated liposomal doxorubicin resistance and the prognosis of treated patients.^23–25^ The precise mechanistic functions of TOP2A in OC, however, have yet to be fully elucidated.

The phosphatidylinositol 3-kinase (PI3K)/AKT/mTOR pathway is an important regulator of cellular proliferative, angiogenic, metabolic, and survival activities, shaping transcription, translation, growth, and other key processes. The serine/threonine kinase AKT, which includes three subtypes (AKT1/2/3), can be stimulated in response to the excessive production of particular cytokines and growth factors, whereupon it can activate or suppress the expression or activity of a range of downstream targets, including NF-κB, Bcl2, Caspase-9, glycogen synthase kinase 3 (GSK3), and CyclinD1, regulating proliferative, migrator, and apoptotic activity.^26^ As a downstream target of AKT, mTOR can be directly phosphorylated by this kinase or indirectly activated by the tuberous sclerosis 1/2 (TSC1/2) complex, upregulating a range of transcription factors that modulate protein synthesis, motility, growth, and survival.^27^

Altered signaling via the PI3K/Akt/mTOR pathway has been reported to shape the development of many forms of cancer. Indeed, gene amplifications or mutations in components of this pathway have been reported in over 70% of breast and colorectal cancer patients.^28,29^ The same is also true in OC, where the activation of the PI3K/Akt/mTOR pathway is related to more aggressive disease, chemoresistance, and poor prognostic outcomes.^30^ Lyu et al. observed the ability of TOP2A to promote gallbladder tumor proliferative and metastasis via the PI3K/AKT/mTOR signaling pathway.^31^ Does TOP2A similarly drive OC progression via the modulation of PI3K/Akt/mTOR signaling?

Here, a series of experiments was conducted in an effort to clarify the mechanistic role of TOP2A in OC. Through these analyses, TOP2A was found to be upregulated in this form of cancer and associated with poor prognostic outcomes. Mechanistically, both *in vitro* and *in vivo* analyses indicated that TOP2A is capable of regulating OC cell proliferation via the regulation of AKT/mTOR signaling pathway.

## Results

### Screening of differential gene expression in OC in publicly available datasets

**Figure 1.**
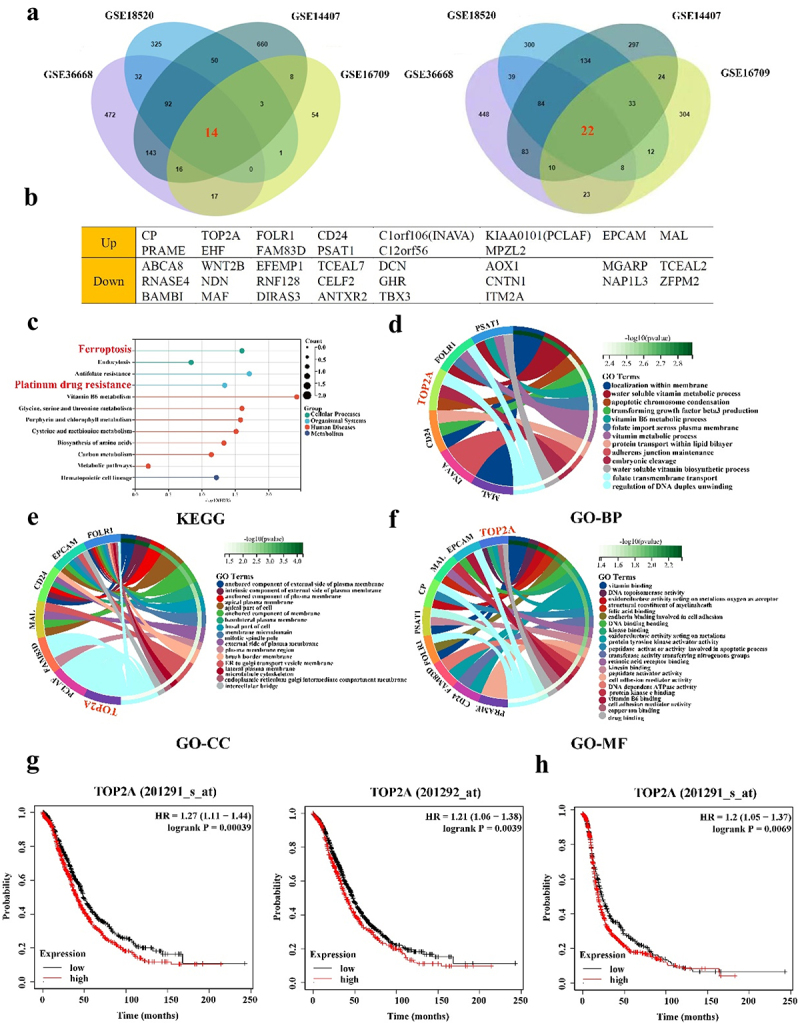
Screening and analysis of DGEs in OC. (a) 14 DEGs were up-regulated and 22 DEGs were down-regulated in four datasets (log2-fold change > 2, *p* < .05). (b) The 14 up-regulated genes and 22 down-regulated genes. (c-f) KEGG, GO-BP, GO-CC and GO-MF analysis of up-regulated genes. (g) High TOP2A expression in dataset 201,291-s-at and 201,292-at was associated with poor overall survival (OS) of OC patients. (h) High TOP2A expression in dataset 201,291-s-at was associated with poor progression-free survival (PFS) of OC patients.

Four Gene Expression Omnibus (GEO) datasets were initially analyzed to assess patterns of differential gene expression in OC (Figure 1a). A total of 36 differentially expressed genes (DEGs) were identified through the screening of these four databases, including 14 and 22 that were upregulated and downregulated in OC, respectively (Figure 1b).

Functional analyses of the upregulated DEGs were next conducted, revealing their enrichment in Kyoto Encyclopedia of Genes and Genomes (KEGG) pathways related to ferroptosis, platinum drug resistance, and metabolism (Figure 1c). These genes were also enriched in Gene Ontology (GO) terms including apoptotic chromosome condensation, regulation of DNA duplex unwinding, DNA topoisomerase activity, and cadherin binding involved in cell adhesion terms (Figure 1d–f). For corresponding details regarding KEGG and GO analysis results for downregulated DEGs, see Figure S1b-e.

To further examine the potential roles of upregulated DEGs and their association with OC patient outcomes, survival analyses were next performed. CP, FOLR1, CD24, C1orf106, KIAA0101, CO-17A, PRAME, and PSAT1 levels were not significantly related to OC patient OS (Figure S2A). However, low expression levels of EHF (*p* = .009) and high expression levels of MAL (*p* < .001), FAM83D (*p* < .001), and C12orf56 (*p* = .017) were associated with poor patient OS (Figure S2B-C). TOP2A (201291-s-at, *p* < .001; 201292-at, *p* = .0039) levels were associated with significant reductions in patient overall survival (OS) (Figure 1g), with high TOP2A (*p* = .0069) expression also being associated with poorer progression-free survival (PFS) in OC patient (Figure 1h). Given these promising results, TOP2A was chosen as a target for further research.

### TOP2A is overexpressed in patients with OC

**Figure 2.**
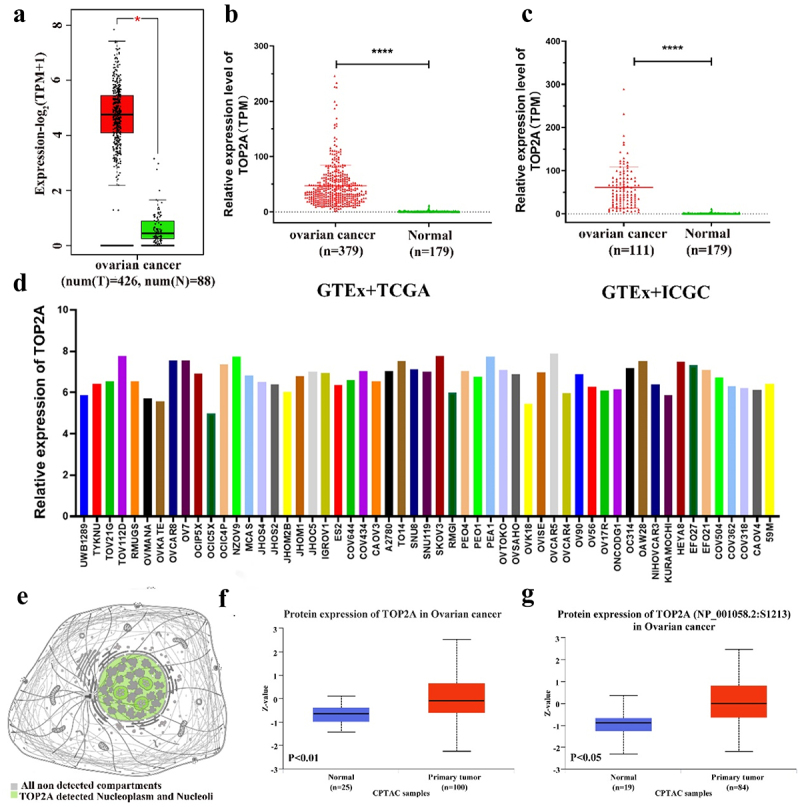
TOP2A expression levels in OC. (a) TOP2A mRNA expression levels were significantly increased in OC patients in the GEPIA database. (b-c) TOP2A mRNA expression levels in OC patients were significantly increased in GTEx combined with TCGA database and in GTEx combined with ICGC database. (d) Expression of TOP2A mRNA in different OC cell lines in CCLE database. (e) The localization of TOP2A protein in cells was obtained through the subcell section of the human protein atlas. (f) Total TOP2A protein expression was increased in OC tissues compared with normal tissues. (g) Compared with normal tissues, the expression of p-TOP2A protein was enhanced in OC tissues. *p < .05, ****p < .0001.

Analyses of the Gene Expression Profiling Interactive Analysis (GEPIA) database next confirmed the upregulation of TOP2A at the mRNA level in patients with OC compared to healthy tissue samples (Figure 2a), while the Genotype-Tissue Expression (GTEx)-based analysis of The Cancer Genome Atlas (TCGA) and International Cancer Genome Consortium (ICGC) datasets indicated that the levels of TOP2A expression in OC were significantly enhanced compared to those in healthy tissue samples (Figure 2b–c). Moreover, pronounced differences in the mRNA levels of TOP2A were detected in different OC cell lines (Figure 2d).

At the protein level, TOP2A was found to primarily localize to the nucleoplasm and nucleolar compartments (Figure 2e), with the Clinical Proteomic Tumor Analysis Consortium (CPTAC) database revealing pronounced increases in the levels of this protein in tissue samples from patients with OC (Figure 2f). When levels of phosphorylated TOP2A (p-TOP2A) were compared between tumors and normal tissues, marked increases in p-TOP2A (S1213, S1106, S1393, S1247) protein levels were detected in OC tissues (Figure 2g, Figure S2D). Immunohistochemical staining further confirmed the enhanced expression of TOP2A in OC patient tumors (Figure S2E). Overall, these findings provide strong evidence for the overexpression of TOP2A at the mRNA and protein levels in OC tumors.

### Validation of the expression and prognostic implications of TOP2A in OC

**Figure 3.**
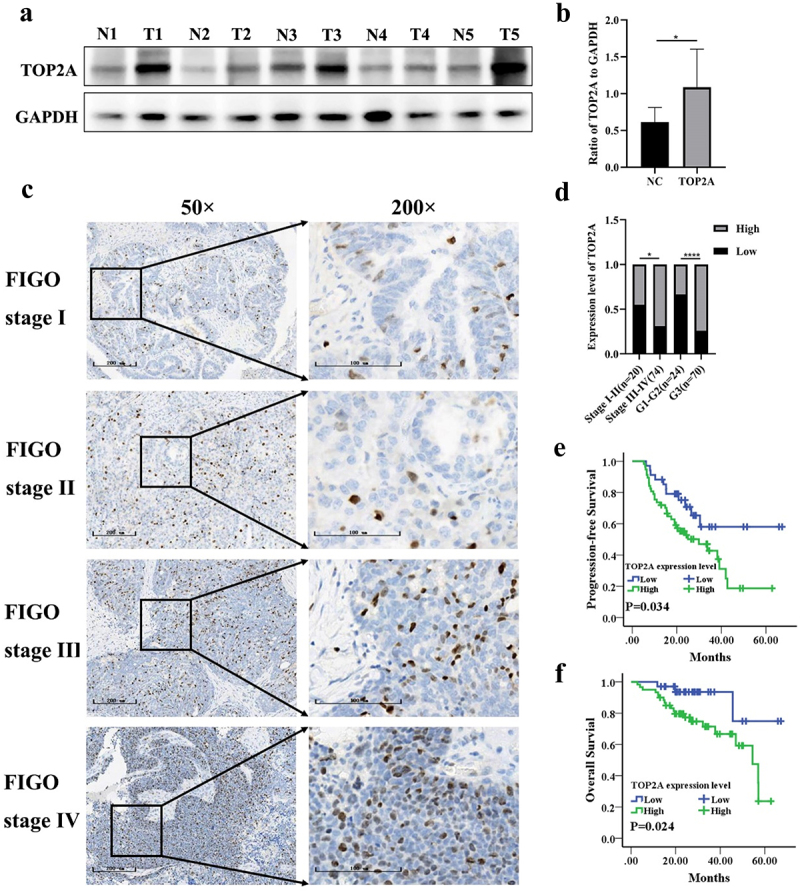
To investigate the expression and prognostic significance of TOP2A in OC by clinical samples. (a-b) TOP2A protein expression level was increased in 5 OC tissues compared with 5 normal tissues by western blotting. (c) The immunohistochemistry atlas of different FIGO stages of 94 ovarian cancer tissues. 50× and 200× (partial enlargement) microscopic views were shown. (d) TOP2A protein expression was enhanced with the increase of FIGO stage and histological grade. (e) TOP2A high expression is associated with poor PFS in OC patients(*p* = .034). (f) TOP2A high expression is associated with poor OS in OC patients(*p* = .024). *p < .05, ****p < .0001.

Next, clinical samples were leveraged in an effort to validate the abovementioned results. When Western blotting was conducted using samples of tumor and normal tissue, TOP2A overexpression was confirmed in OC tumors (Figure 3a–b). Additionally, the results of IHC staining showed that 34 and 60 of the analyzed patient samples respectively exhibited low and high staining for TOP2A (Figure 3c), and TOP2A expression tended to increase with increasing FIGO stage and histological grade (Figure 3c–d).

The association between TOP2A expression in OC and a range of clinicopathological factors was next examined, revealing it to be associated with late FIGO stage and high histological grade, whereas it was unrelated to age or lymph node status (Table 1).

**Table 1.** The relationship between TOP2A expression and clinicopathological features in ovarian cancer.

	TOP2A expression level			
Variable	Low (%)	High (%)	*χ*^*2*^*/t*	*P*-value
Total Number	34 (36.2)	60 (63.8)		
Age	62.32 ± 9.91	58.85 ± 8.55	1.786*	0.077
FIGO stage			3.902^#^	0.048
I-II	11 (55.0)	9 (45.0)		
III-IV	23 (31.1)	51 (68.9)		
Histology grade			12.983^#^	0.000
G1-G2	16 (66.7)	8 (33.3)		
G3	18 (25.7)	52 (74.3)		
Lymph node metastasis			0.389^#^	0.533
No	17 (39.5)	26 (60.5)		
Yes	17 (33.3)	34 (66.7)		

*Student t-test; ^#^Chi-Squared Test.

Kaplan-Meier curves were also used to assess the prognostic relevance of TOP2A in these patients. Of the TOP2A low expression patients, 11 relapsed and 3 died, whereas 33 and 20 of the TOP2A high expression patients experienced these respective outcomes. The PFS and OS of TOP2A low expression patients was significantly longer than that of TOP2A high expression patients (All *p* < .05, Figure 3e–f).

### TOP2A silencing inhibits malignant progress of OC cells

**Figure 4.**
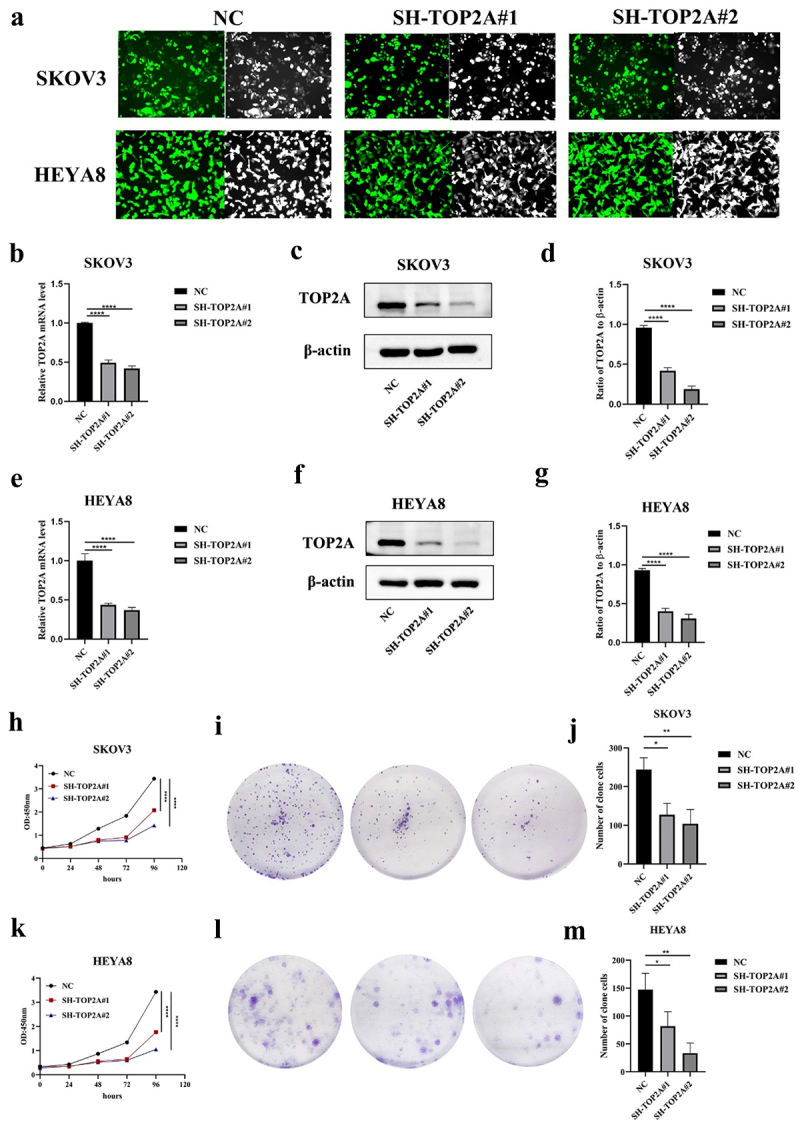
The effect of TOP2A knockdown on OC cell proliferation. (a) The transfection efficiency of SH-TOP2A in the two cell lines was observed by fluorescence microscope. (b-d) TOP2A mRNA and protein levels were significantly decreased after transfection in SKOV3 cells. (e-g) TOP2A mRNA and protein levels were significantly decreased after transfection in HEYA8 cells. (h-j) CCK-8 and colony formation assays suggested that the proliferation ability of SKOV3 cells was decreased after TOP2A knockdown. (k-m) CCK-8 and colony formation assays suggested that the proliferation ability of HEYA8 cells was decreased after TOP2A knockdown. Number of experiments (N) = 3, *p < .05, **p < .01, ***p < .001, ****p < .0001.

To generate cells in which TOP2A was stably knocked down, shRNA constructs were introduced into both SKOV3 and HEYA8 cells, which exhibited high levels of transduction efficiency (Figure 4a). Subsequent qPCR and Western blotting analyses confirmed that TOP2A was successfully knocked down at the mRNA and protein levels compared to negative control group (NC) cells (Figure 4b–g), enabling the utilization of these cells in the next series of experiments.

A CCK-8 assay was then used to evaluate the proliferative activity of these cells, demonstrating that TOP2A silencing reduced the proliferation of both SKOV3 and HEYA8 cells in comparison with NC controls. The same effect was also detected in a colony formation assay. The number of clone cells were 244 ± 30.61, 127.33 ± 29.4 and 104 ± 36.76 in SKOV3 cells, and 147.33 ± 29.01, 81.67 ± 26.16 and 33.33 ± 18.23 in HEYA8 cells, suggesting that the knockdown of TOP2A resulted in the impairment of OC cell proliferative activity (Figure 4h–m).

**Figure 5.**
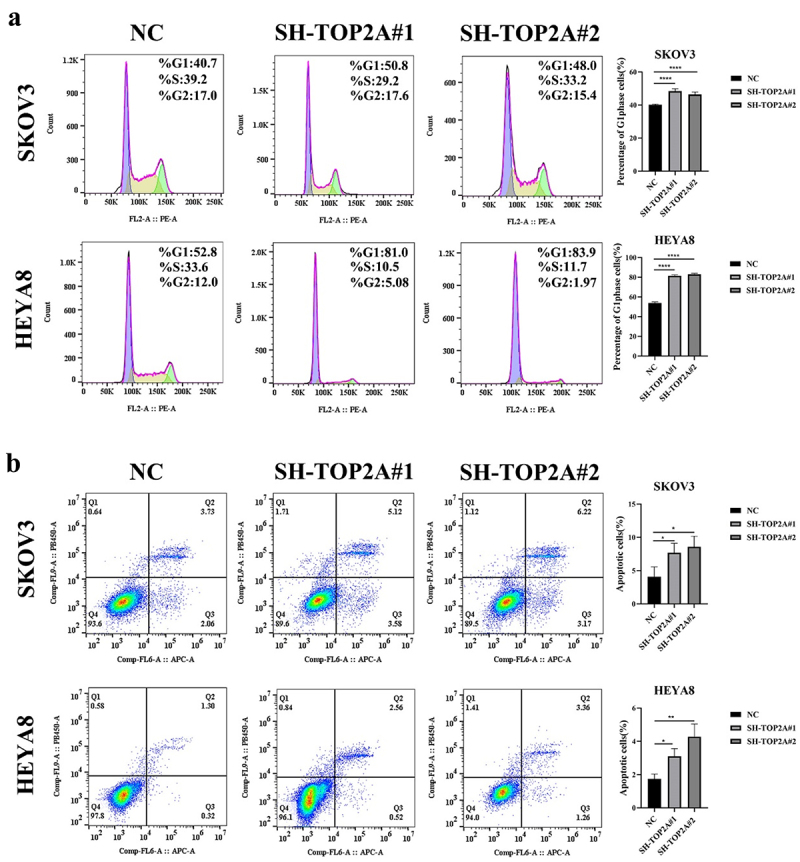
The effect of TOP2A knockdown on OC cell cycle and apoptosis. (a) TOP2A knockdown increased the proportion of cells in G1 phase in both OC cell lines. (b) TOP2A knockdown promoted apoptosis in both OC cell lines. *N* = 3, *p < .05, **p < .01, ****p < .0001.

A flow cytometry approach was further employed to examine changes in apoptosis and cell cycle progression following the silencing of TOP2A. TOP2A knockdown was associated with an increase in the frequency of cells in the G1 phase relative to the NC group. The results were 40.2 ± 0.37, 48.38 ± 1.52 and 46.44 ± 1.49% in SKOV3 cells, and 53.90 ± 1.17, 81.42 ± 1.04 and 82.98 ± 1.45% in HEYA8 cells, consistent with higher rates of G1 phase cell cycle arrest in the absence of normal TOP2A expression (Figure 5a). Consistent with these findings, both down-regulation groups (SH-TOP2A) exhibited increases in the frequencies of early and late apoptotic cells compared to control cells (Figure 5b). The results were 4.09 ± 1.48, 7.69 ± 1.43 and 8.01 ± 1.36% in SKOV3 cells, and 1.74 ± 0.30, 3.11 ± 0.46 and 4.28 ± 0.76% in HEYA8 cells, emphasizing the ability of TOP2A knockdown to induce OC cell apoptotic death.

### TOP2A silencing modulates the expression of proteins related to cell proliferation, cell cycle and apoptosis

**Figure 6.**
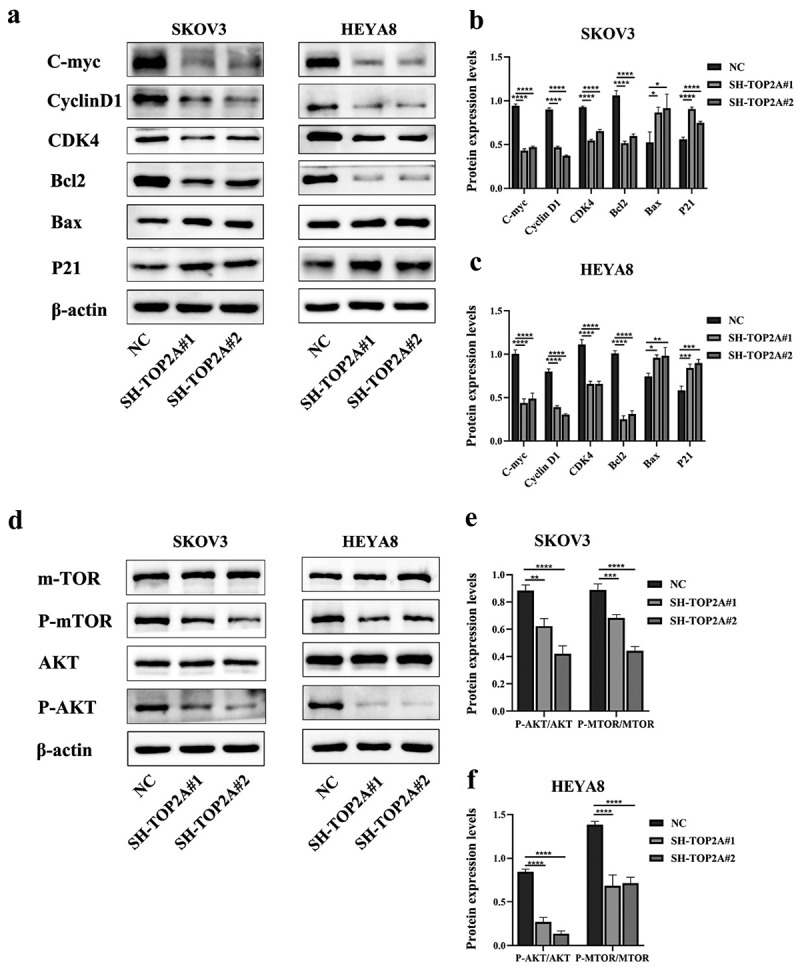
The effect of TOP2A knockdown on related proteins. (a-c) after TOP2A knockdown, the expression levels of C-myc, cyclin D1, CDK4 and Bcl2 were decreased, while the expression levels of P21 and Bax were increased. (d-f) after TOP2A knockdown, the expression levels of p-AKT/AKT and p-mTOR/mTOR were decreased. *N* = 3, *p < .05, **p < .01, ***p < .001, ****p < .0001.

Western blotting was next conducted to assess protein expression changes, revealing that TOP2A knockdown was associated with lower levels of C-myc, cyclin D1, CDK4, and the anti-apoptotic protein Bcl2, whereas P21 and the pro-apoptotic protein Bax were upregulated in the same OC cells (Figure 6a–c).

Lower levels of p-AKT and p-mTOR were also detected in both SH-TOP2A groups when normalized to total AKT and mTOR levels relative to the NC group (Figure 6d–f). This finding thus suggested that the ability of TOP2A to influence OC cell proliferative activity, cell cycle progression, and apoptotic death may be mediated via the AKT/mTOR pathway.

### In vitro validation of the mechanisms whereby TOP2A influences OC cell behavior

**Figure 7.**
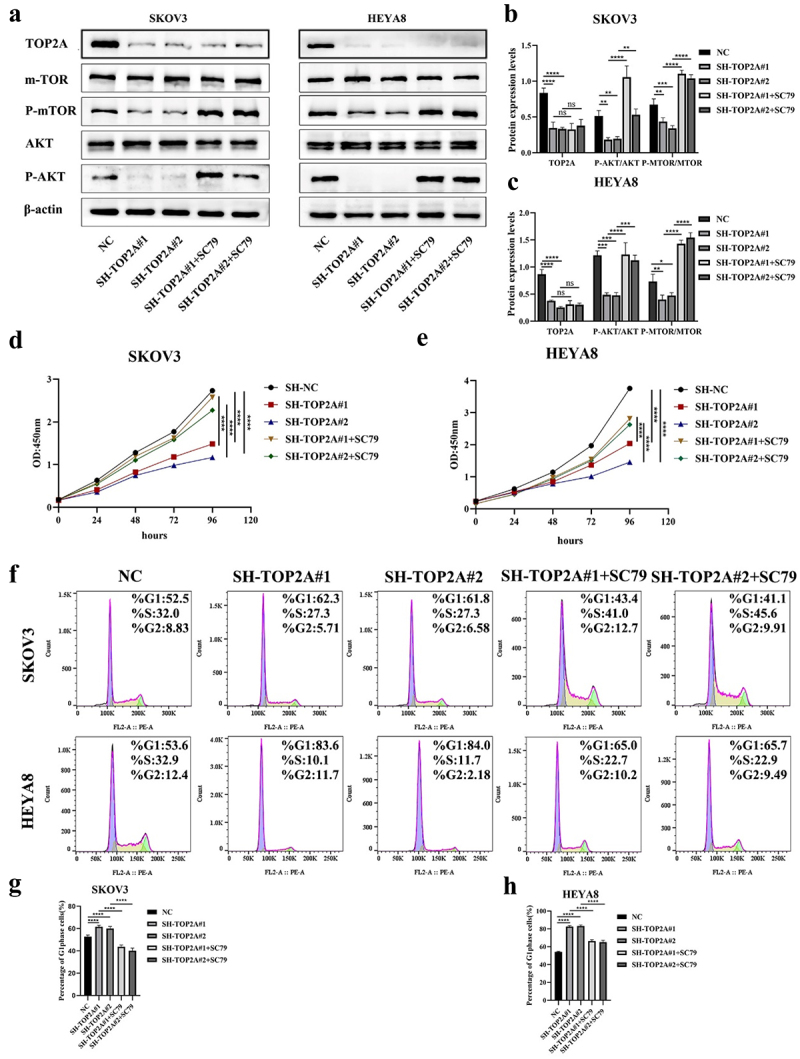
Rescue experiments were conducted to verify the mechanism of TOP2A regulating OC cell behavior. (a-c) after SC79 treatment of cells in the SH-TOP2A group, WB results suggested that the levels of p-AKT/AKT and p-mTOR/mTOR were increased. (d-e) CCK-8 assays showed that the cell proliferation ability of the two SH-TOP2A groups was enhanced after SC79 treatment. (f-h) flow cytometry confirmed that SC79 induced the G1-S phase progression of the cell cycle in both SH-TOP2A groups. *N* = 3, *p < .05, **p < .01, ***p < .001, ****p < .0001.

A series of rescue experiments were next conducted to validate the ability of TOP2A to influence OC cell biology via AKT/mTOR pathway regulation. When cells in which TOP2A had been silenced were treated with the AKT activator SC79, a 4 μg/mL dose of this drug was found to activate AKT/mTOR pathway signaling as evidenced by higher p-AKT and p-mTOR levels detectable through Western blotting (Figure 7a–c).

In subsequent CCK-8 assays, SC79 treatment was found to enhance the proliferation of TOP2A-knockdown OC cells (Figure 7d–e). Flow cytometry further confirmed that AKT/mTOR pathway activation in this model system was associated with a reduction in the frequency of cells in the G1 phase relative to pretreatment levels. The results were 52.9 ± 1.36, 61.7 ± 1.2, 60.06 ± 1.9, 43.66 ± 1.64 and 40.2 ± 2.3% in SKOV3 cells, and 54.26 ± 0.65, 82.7 ± 1.12, 83.18 ± 1.35%, 66.34 ± 1.57 and 65.16 ± 1.94% in HEYA8 cells, consistent with the ability of SC79 to induce the G1-S phase progression of the cell cycle, thereby facilitating the proliferation of OC cells through AKT/mTOR signaling activity (Figure 7f–h).

**Figure 8.**
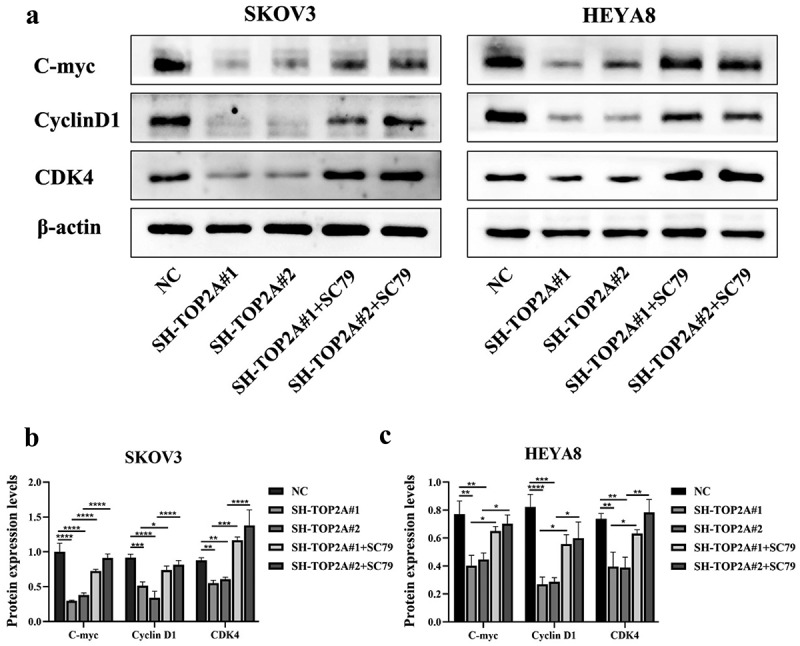
Expression of related proteins after activation of AKT/mTOR pathway. (a-c) the protein levels of C-myc, CyclinD1 and CDK4 in the SH-TOP2A#1+SC79 and SH-TOP2A#1+SC79 groups were higher than those before treatment of SC79. *N* = 3, *p < .05, **p < .01, ***p < .001, ****p < .0001.

AKT/mTOR pathway activation was associated with increased C-myc, CyclinD1, and CDK4 protein levels in both TOP2A knockdown groups relative to pretreatment levels, as assessed via WB (Figure 8a–c).

### To verify the effect of TOP2A knockdown on the growth of OC cells in vivo

**Figure 9.**
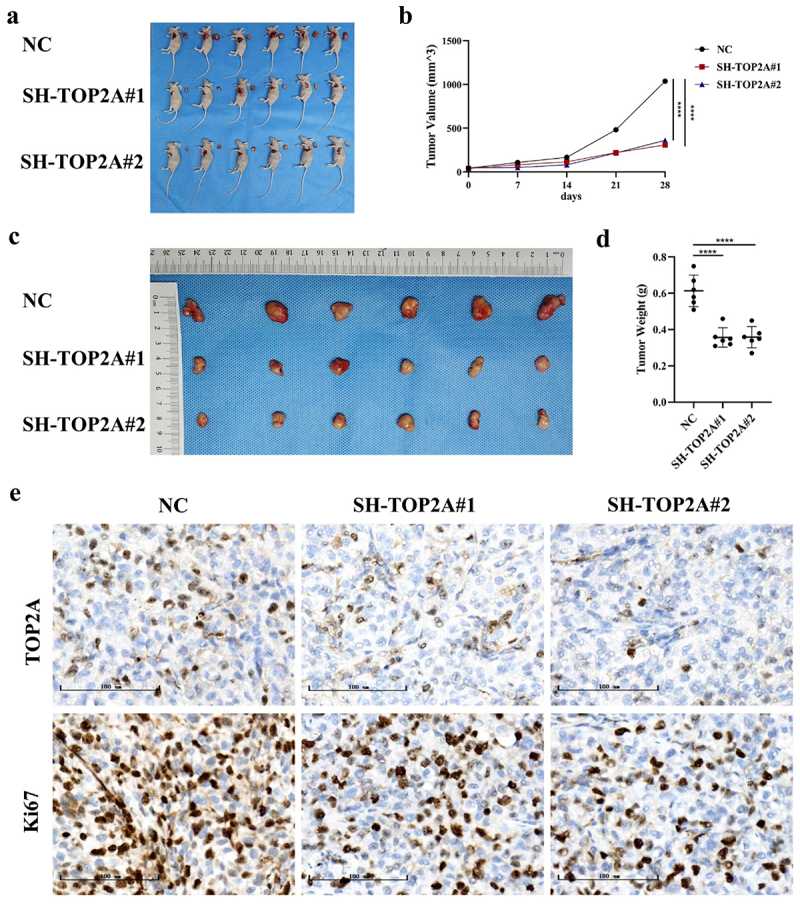
The effect of TOP2A knockdown on the growth of OC cells in vivo. (a-d) TOP2A knockdown significantly decreased the HEYA8 cell-derived tumor volume and weight. (e) IHC staining revealed reductions in TOP2A and ki-67 levels in both SH-TOP2A groups relative to tumors from NC group. ****p < .0001.

After establishing and monitoring a murine model of xenograft OC tumor growth, animals were sacrificed, and tumors were resected, weighed, and imaged. These analyses revealed that silencing TOP2A significantly decreased the HEYA8 cell-derived tumor volume and weight in these animals (Figure 9a–d). IHC staining of tumors from these animals also revealed reductions in TOP2A and Ki-67 levels in both SH-TOP2A groups relative to tumors from NC animals (Figure 9e), with these expression levels being significantly correlated with one another (r_s_ = 0.791, *p* < .001).

## Discussion

Effectively treating recurrent OC remains a persistent difficulty facing gynecological oncologists. While TOP2A inhibitor is widely deployed as treatments for platinum-resistant recurrent OC cases, the efficacy remains limited. Data have shown that oral etoposide in the treatment of recurrent ovarian cancer, the objective response rate (ORR) was 20.4% (range, 0 to 30.5%).^32^ However, one clinical study showed that Oral etoposide combined with Apatinib in the treatment of platinum-resistant or platinum-refractory ovarian cancer patients, the ORR was as high as 54%,^10^ showing the broad prospects of drug combination therapy. In our study, we found that TOP2A expression was enhanced in OC tissues and correlated with FIGO stage, histological grade and poor prognosis. Based on our *in vitro* and *in vivo* studies, we demonstrated that TOP2A promoted OC proliferation via Akt/mTOR pathway.

Owing to the widespread applications of bioinformatics techniques in cancer, some databases have been established in recent years including the TCGA,^33^ SEER,^34^ and GEO databases.^35^ These resources offer a wealth of data related to tumor diagnosis and treatment. Here, public dataset analyses suggested that TOP2A was highly expressed at the mRNA and protein levels in OC tumor tissues (Figure 2), with such expression being related to poor OC patient prognostic outcomes such that it was selected as a target for further research (Figure 1g–h).

Subsequent clinical analyses confirmed the expression of high TOP2A levels in OC patient samples (Figure 3a,b), with its upregulation being correlated with both more advanced FIGO staging and higher histological grade (Figure 3c,d), supporting a potential role for TOP2A as an oncogene in this form of cancer. TOP2A was also found to be associated with worse patient PFS and OS (Figure 3e,f), in line with the online database analyses. Some prior publications have also confirmed the oncogenic importance of TOP2A,^18,20–22^ providing greater support for these results.

To validate the above findings and to explore their underlying mechanisms, OC cell lines in which TOP2A had been stably knocked down were next constructed (Figure 4a–g). The silencing of TOP2A in these cells was found to be associated with the inhibition of OC cell proliferation (Figure 4h–m), G1 phase arrest (Figure 5a), and the induction of apoptotic death (Figure 5b), thus aligning well with other recent publications. For example, Liu et al. noted a link between TOP2A and tumor cell proliferation related to MDM4 interactions,^36^ while Zhang et al. demonstrated that knocking down TOP2A in colon cancer cells led to the inhibition of their proliferative activity and invasivity.^17^ Cui et al. further noted the ability of miR-27a-5p to inhibit gastric cancer cell proliferation, invasivity, and migration while inducing apoptotic death as a consequence of a reduction in TOP2A expression.^37^ The present data thus extend these results to OC, emphasizing the ability of TOP2A to promote proliferative activity in this cancer setting.

Cyclin D1 is closely involved in cell cycle regulation, and it is classically involved in the regulation of oncogenesis through its ability to promote the G1-S phase transition by binding interactions with CDK4.^38^ Indeed, many studies have affirmed the importance of this CyclinD1/CDK4 complex in the regulation of cell cycle progression. In renal cells, for example, Dozier et al. demonstrated the ability of the Cyclin D1/CDK4/6 complex to directly phosphorylate CDC25A during the G1 phase, thereby regulating its stability.^39^ Ding et al. separately found that SOCS1 was capable of disrupting the G1-S transition in hepatocellular carcinoma via destabilizing the nuclear Cyclin D1/CDK4 complex and thus interfering with tumor cell proliferative activity.^40^ Regulatory roles for this complex have also been confirmed in bladder cancer, pancreatic cancer, triple-negative breast cancer, and other malignancies.^41–43^ In OC, CyclinD1 has also been suggested to contribute to proliferation, although the CyclinD1/CDK4 complex has been linked.^44,45^ The cyclin-dependent kinase inhibitor P21 can reportedly interact with and inhibit the activity of the cyclin-CDK2, -CDK1, and -CDK4/6 complexes, thereby regulating the G1-S phase and inhibiting its activity, thus regulating the cell cycle in G1 and S phase.^46^ Proteins encoded by members of the c-Myc oncogene family are commonly activated in a wide range of tumors wherein they influence growth and malignancy.^47^ Here, C-myc, CyclinD1, CDK4, and the anti-apoptotic protein Bcl2 were all downregulated following TOP2A knockdown, whereas P21 and the pro-apoptotic protein Bax were upregulated (Figure 6a–c). This suggests a decrease in the proliferative ability of OC cell lines, with TOP2A silencing resulting in G1 phase arrest and apoptotic cell death, which was supported by cellular experiments. As such, these findings indicated that TOP2A influences proliferation, apoptosis, and progression through the cell cycle by regulating protein targets including C-myc, the CyclinD1/CDK4 complex, P21, Bcl2, and Bax.

The PI3K/AKT/mTOR pathway is central to the oncogenic development and progression of OC, regulating a diverse array of targets to shape metastatic, proliferative, apoptotic, and other activities.^30,48,49^ Strikingly, TOP2A was herein found to be associated with changes in the expression of downstream targets including P21, cyclin D1, Bcl2, Bax, and others, with corresponding reductions in the levels of p-AKT and *p*- mTOR (Figure 6). Based on these results, TOP2A was hypothesized to influence the AKT/mTOR pathway to regulate OC progression, as confirmed in subsequent rescue experiments.

The AKT agonist SC79 is capable of phosphorylating and activating a range of AKT subtypes such that it is widely employed in studies focused on the PI3K/AKT/mTOR signaling pathway.^50,51^ Here, an increase in p-AKT and p-mTOR levels was noted in TOP2A-knockdown cells following SC79 treatment (Figure 7a–c), which rescued the proliferative ability of these OC cells and promoted the G1-S phase cell cycle transition (Figure 7d–h). These data further confirmed the ability of TOP2A to modulate C-myc and CyclinD1/CDK4 complex expression levels in an AKT/mTOR pathway-dependent fashion, ultimately promoting OC cell proliferation (Figure 8a–c).

*In vitro* analyses revealed that knocking down TOP2A strongly impacted the proliferation of cells, while GO enrichment analyses of RNA-seq results confirmed the importance of this gene in the context of OC cell proliferation (Figure S3). To validate these findings *in vivo*, a murine xenograft model was established in which knocking down TOP2A was confirmed to inhibit tumor growth, and the strong correlation observed between TOP2A and Ki-67 further confirmed the close relationship between this gene and cellular proliferation.

Although Withoff et al. and Faggard et al. have analyzed the expression and prognostic value of TOP2A in OC, they only conducted their study through clinical data.^52,53^ And in our study, through a combination of bioinformatics techniques and preclinical model systems, OC patient tissues were herein found to express elevated levels of TOP2A, with such upregulation being closely related to poor prognostic outcomes. Mechanistically, TOP2A was further confirmed to be capable of regulating the AKT/mTOR pathway to OC progression. Therefore, it is worth exploring whether TOP2A inhibitors and AKT inhibitors have a combined effect on OC. At the same time, one study has confirmed that pegylated liposomal doxorubicin (PLD) combined with olaparib has significant activity in platinum-resistant ovarian cancer.^54^ PLD and etoposide are both TOP II inhibitors, and whether etoposide combined with olaparib is effective for platinum-resistant ovarian cancer is also worthy of further exploration. Even so, this study is subject to some limitations. For one, the sample size included in this study is limited. Additional large-scale studies will be essential to confirm this relationship and expand upon these findings.

In conclusion, TOP2A was herein confirmed to serve as an oncogenic factor and prognostic biomarker in OC, driving disease progression via the activation of the AKT/mTOR pathway. These results may additionally provide a theoretical foundation for efforts to develop novel combination therapies for individuals suffering from recurrent OC.

## Materials and methods

### Data collection

Initially, the GEO database (https://www.ncbi.nlm.nih.gov/gds) was searched in an effort to identify datasets related to OC, with the GSE36668, GSE18520, GSE14407, and GSE16709 microarray datasets ultimately being downloaded for analysis. These datasets were analyzed with the GEO2R tool, and DEGs were identified based on a log2 (fold-change) > 2 and a Benjamini & Hochberg *p* value < 0.05. Volcano plots of these DEGs were generated using OmicStudio (https://www.omicstudio.cn/index) (Figure S1A), and only those DEGs appearing in all datasets were considered high-confidence DEGs.

DEG enrichment in particular GO terms and KEGG pathways was evaluated with Sangerbox (http://SangerBox.com/Tool), with *p* < .05 as the cutoff to define significance. Relationships between upregulated DEGs and OC patient prognostic outcomes were additionally examined with Kaplan‒Meier Plotter (https://kmplot.com/analysis/).^55^

To further evaluate OC-related patterns of TOP2A expression, the GEPIA2 database was utilized (http://gepia2.cancer-pku.cn/#general),^56^ as were data from the GTEx, TCGA, and ICGC projects pertaining to TOP2A levels in OC. Row data were normalized through TPM transformation. TOP2A expression data for the OC cell line were further accessed through the CCLE database (https://sites.broadinstitute.org/ccle/),^57^ and the results were analyzed with GraphPad Prism 9.0 (CA, USA).

TOP2A protein localization within cells was interrogated with the Human Protein Atlas (https://www.proteinatlas.org/) Cell Atlas module, and immunohistochemical (IHC) staining sections from control and OC tumor tissues were also obtained. The CPTAC module of UALCAN (http://ualcan.path.uab.edu/analysis-prot.html) was also leveraged to assess the protein levels of both total TOP2A and p-TOP2A.^58^ *p* < .05 was the cutoff used to define significance.

### Patient sample collection

Samples of fresh tissue were obtained from 5 OC patients and 5 individuals with benign tumors undergoing surgery in the Department of Obstetrics and Gynecology of Tianjin Medical University General Hospital from May 2022 through August 2022. After collection, these tissues were snap-frozen with liquid nitrogen followed by storage at −80°C. The expression level of TOP2A was detected by Western blotting.

In total, this study enrolled 94 OC patients from the General Hospital of Tianjin Medical University between January 2018 and November 2022. Eligible patients included: (1) individuals undergoing surgery, (2) individuals diagnosed with epithelial OC through routine pathological analyses, and (3) individuals undergoing standardized postoperative adjuvant treatment. Patients were excluded if they: (1) had been pathologically diagnosed with non-epithelial OC, (2) underwent preoperative chemotherapy or radiotherapy, or (3) exhibited other malignancies. Tissue sections were prepared for IHC analyses to explore the expression level of TOP2A in tumor tissues. Two experienced pathologists used a standardized scoring system to evaluate all staining results, and complete pathological and clinical data were available for all patients. Telephone-based follow-up was conducted through September 1, 2023. The Ethics Committee of Tianjin Medical University General Hospital provided approval for this study (Ethical No. IRB2019-WZ-044), and all patients gave written informed consent.

### Hematoxylin-eosin staining (HE)

Samples of fresh tissue were fixed for 48 h in 10% neutral formalin to ensure that proteins were denatured and coagulated, after which tissues were dehydrated with ethanol and treated with xylene for 30 minutes to achieve transparency. These tissues were then embedded in paraffin, cut into 4 μm sections, treated with xylene to remove the paraffin, rehydrated with an ethanol gradient, stained with hematoxylin and eosin (H&E), treated with neutral gum, sealed, and fixed for microscopic analysis and imaging.

### Immunohistochemistry (IHC)

IHC analyses were performed as described in prior studies.^59^ After probing overnight with primary anti-TOP2A (1:5000, ab52394, Abcam) at 4°C, tissue samples were probed for 15 min with a secondary antibody (PV9001, ZSGH-Bio) at room temperature. TOP2A expression was then assessed based on the intensity and frequency of staining, with two experienced pathologists independently scoring these samples in a double-blinded manner.

Staining intensity (0: no staining, 1: weak, 2: moderate, 3: strong) and the percentage of tumor cells stained for TOP2A (0: ≤ 5%, 1: 6–25%, 2: 26–50%, 3: 51–75%, 4: > 75%) were scored and summed to yield a comprehensive score ranging from 0 to 7. Based on the median score for each sample, which was used as a cutoff value, a score of 0 was indicative of no expression, while values below and above this value were used to define low and high levels of expression, respectively. These latter two sample groups were combined to identify the number of samples positive for TOP2A expression to permit downstream statistical analyses.

### Cell culture and cell transfection

The human HEYA8 and SKOV3 OC cell lines were obtained from the American Type Culture Collection and routinely cultured in RPMI-1640 containing 10% FBS (10099141C, Gibco) and 1% penicillin – streptomycin in a 37°C, 5% CO_2_ incubator.

RNA lentiviruses were purchased from the company (GENE, Shanghai, China). And a standard approach was used to perform lentiviral transduction of these cells with the packaged NC vector and ShRNA-TOP2A vector. Initially, the NC group and down-regulation group were set up, and HEYA8 and SKOV3 cells were plated in 6-well plates (2 × 10^5^/well) and cultured at 37°C for 24 hours. Then the volumes of NC and ShRNA-TOP2A lentiviruses were calculated according to the number of cells, after which the lentivirus was added to these cells in 1 mL of media containing an infection enhancer, and then added to the corresponding cells, followed by incubation for 16 h at 37°C, with media then being exchanged for fresh complete media. Fluorescence microscopy was used to detect successful cellular transduction at 48–72 h post-transduction based on the presence of green fluorescence, and puromycin was used to select for stably transduced cells. These cells were then expanded for RT-qPCR and Western blotting analyses aimed at confirming successful TOP2A knockdown.

### RT‒qPCR

TOP2A levels in OC cell lines were analyzed via RT-qPCR. After utilizing TRIzol to extract total RNA from these cells and confirming that OD260/280 values were within the appropriate range, a RevertAid First Strand cDNA Synthesis Kit was utilized for cDNA preparation. Next, qPCR analyses were performed with the PowerUP^TM^ SYBR^TM^ Green Master Mix and the 7500 Real-time PCR Instrument, enabling the measurement of relative TOP2A levels via the 2^−ΔΔCT^ method. β-actin served as a normalization control. The sequences of primer used for this study were as follows. β-actin, F:5′-TGGCACCCAGCACAATGAA-3′, R:5′-CTAAGTCATAGTCCGCCTAG AAGC-3′. TOP2A, F:5′- ACCATTGCAGCCTGTAAATGA-3′, R:5′-GGGCGGAGC AAAATATGTTCC-3′.

### Western blotting (WB)

After separating proteins via 8% of 12% SDS-PAGE, they were transferred onto activated PVDF membranes before blocking with 5% skim milk for 2 h. Blots were subsequently probed overnight at 4°C with primary antibodies, washed thrice with TBST, and then probed for 1 h with an appropriate secondary antibody. After further washing with TBST, an enhanced chemiluminescent development reagent was used to detect proteins on these blots, which were imaged with a chemiluminescence imaging system (BLT, Guangzhou, China). ImageJ was used for the densitometric analysis of these samples, and GraphPad Prism 9.0 was used to analyze the resultant data. All analyses were repeated in triplicate. The antibodies used for this study are presented in Table S1.

### CCK-8 assays

Cells were collected while in the logarithmic phase of growth and suspended at 2 × 10^4^ cells/mL prior to plating in 96-well plates (100 μL/well), with five replicates for each treatment group. These cells were cultured for 0, 24, 48, 72, or 96 h in an incubator, followed by the 1:10 dilution of CCK-8 reagent (GK10001, Glpbio) using serum-free media, followed by its addition to these plates (110 μL/well). Following a further 1 h incubation, absorbance at 450 nm was quantified through the use of a microplate reader (Gene Company Limited, China) for each well.

### Colony formation assays

Appropriately transduced cells were harvested in complete media and transferred into 6-well plates (500/well), followed by incubation for 10 days. After the fixation of these colonies with 4% paraformaldehyde (PFA) for 15 min, they were stained for 4 min with 2.5% crystal violet. Colonies were then counted with ImageJ, and the resultant data were analyzed with GraphPad Prism 9.0.

### Flow cytometry

Appropriate cells were suspended at 1 × 10^6^ cells/mL, and 1 mL cell samples were centrifuged prior to staining with an Annexin V-APC/DAPI apoptosis kit (E-CK-A258, Elabscience), with subsequent flow cytometry analyses.

For cell cycle analyses, 1 mL volumes of single-cell suspensions were spun down, resuspended in 500 μL of cold 70% ethanol overnight at 4°C, and the ethanol was then discarded with cells being suspended in cold PBS. After repeating this centrifugation process, cell cycle progression was detected with the DNA Content Quantitation Assay (Cell Cycle) (CA1510, Solarbio) via flow cytometry.

### RNA-seq

Following TOP2A knockdown, changes in transcriptional activity were assessed via RNA-seq. Briefly, RNA was extracted from samples as above, after which sequencing and primary analyses were performed by a company (GENEWIZ, Suzhou, China).

### Drug preparation

Lyophilized SC79 (GC11645, Glpbio), an activator of AKT, was resuspended in 500 µL of DMSO at 20 μg/μL, followed by storage at −20°C for later use.

### Tumor xenograft modeling

The Experimental Animal Ethics Committee of Tianjin Medical University General Hospital approved all animal model studies discussed herein. A subcutaneous xenograft model was established using HEYA8 cells, which were implanted in 5-week-old nude BALB/c female mice that were separated into NC, SH-TOP2A#1, and SH-TOP2A#2 groups (*n* = 6/group). Murine body weight and tumor sizes were assessed once per week. Mice were euthanized via cervical dislocation after 28 days, and tumors were collected, fixed with 10% neutral formalin, and used to conduct IHC and H&E staining. The IHC procedure was described previously and the antibodies used were anti-TOP2A (1:5000, ab52394, Abcam) and anti-Ki67 (1:200, ab16667, Abcam). Tumor volume (V) was calculated as: V= length×width^2^/2, and tumors were monitored to ensure that they did not exceed 15 mm in diameter.

### Statistical analysis

Statistical analyses and figure preparation were performed with SPSS 26.0, ImageJ 1.52, and GraphPad Prism 9.0. Categorical data are given as percentages and compared with chi-square tests, t tests or one-way ANOVAs. The prognostic relevance of TOP2A was assessed with Kaplan-Meier curves and Spearman correlation coefficients. All experiments were conducted a minimum of three times. A two-sided *p* < .05 serves as the cutoff to define significance. (*p < .05, **p < .01, ***p < .001, ****p < .0001).

## Supplementary Material

Supplementary Information_Revised clean.docx

## Data Availability

The datasets used and analyzed during the current study are available from the corresponding author on reasonable request: https://wangyingmei@tmu.edu.cn.
