# Supplementary material for: TOP2A modulates signaling via the AKT/mTOR pathway to promote ovarian cancer cell proliferation
Source: Cancer Biol Ther. 2024 Mar 6;25(1):2325126. doi: 10.1080/15384047.2024.2325126 (PMC10936659; doi:10.1080/15384047.2024.2325126)
Supplement: Supplementary Information_Revised clean.docx [file KCBT_A_2325126_SM2896.docx]

Table S1 Information on antibodies used in Western Blotting

| Antibodies | Catalog number | Company |
| --- | --- | --- |
| TOP2A | ab52934 | abcam |
| GAPDH | 5174S | CST |
| P-mTOR | 5536S | CST |
| mTOR | 2983S | CST |
| P-AKT | 4060S | CST |
| AKT | 4691S | CST |
| C-myc | 1588S | CST |
| Cyclin D1 | 55506S | CST |
| CDK4 | 12790S | CST |
| Bcl2 | 15071S | CST |
| Bax | 41162S | CST |
| P21 | 2947S | CST |
| β-actin | TA-09 | zsbio |


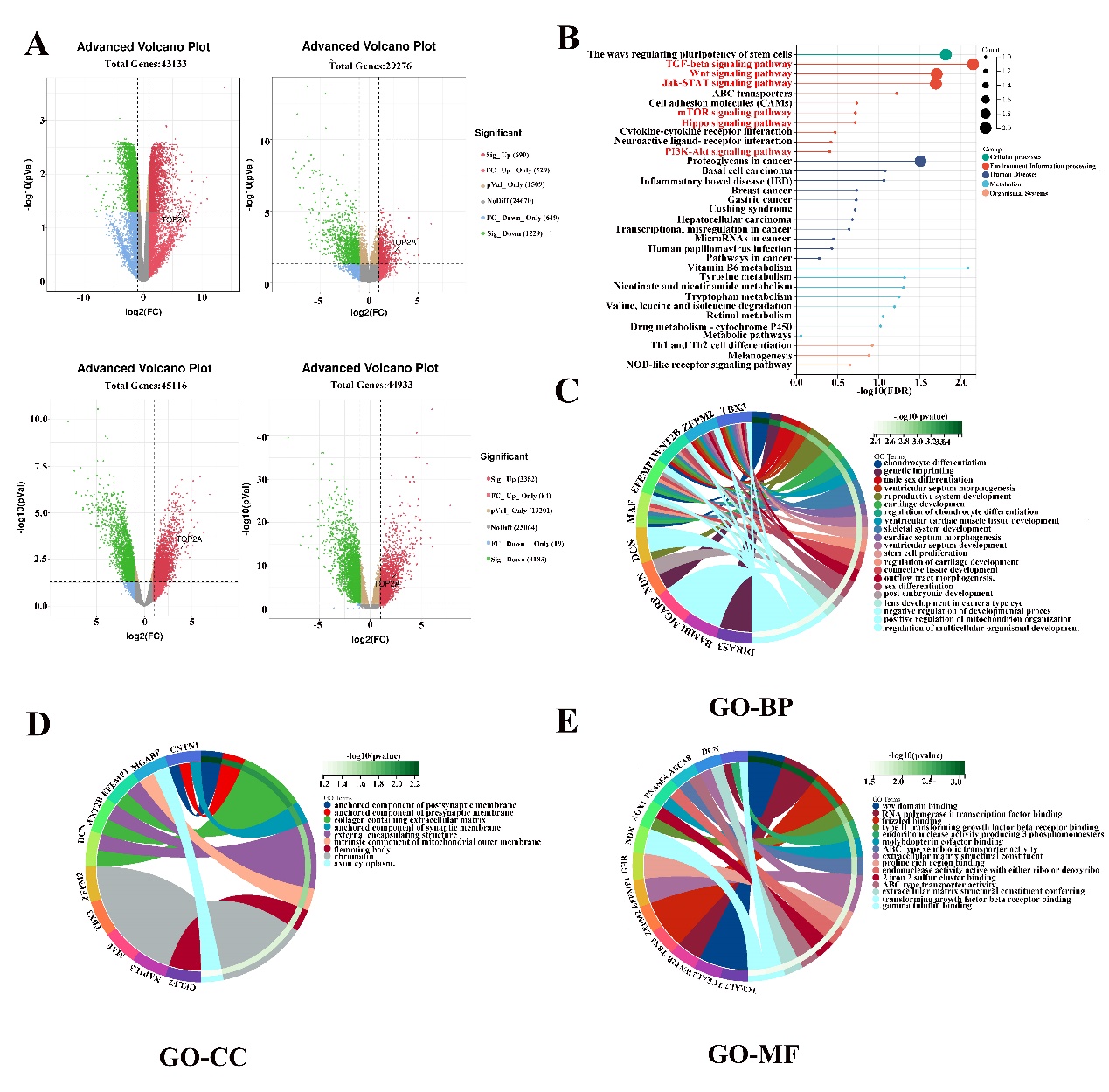
 **Figure S1.** Volcano plots and KEGG, GO analysis of down-regulated genes. (A) The volcano plots of four GEO datasets for differentially expressing mRNAs. Red dots, significantly upregulated genes. Green dots, significantly downregulated genes. TOP2A was labeled. (B) KEGG analysis of 22 down-regulated genes. (C-E) GO-BP, GO-CC and GO-MF analysis of 22 down-regulated genes.


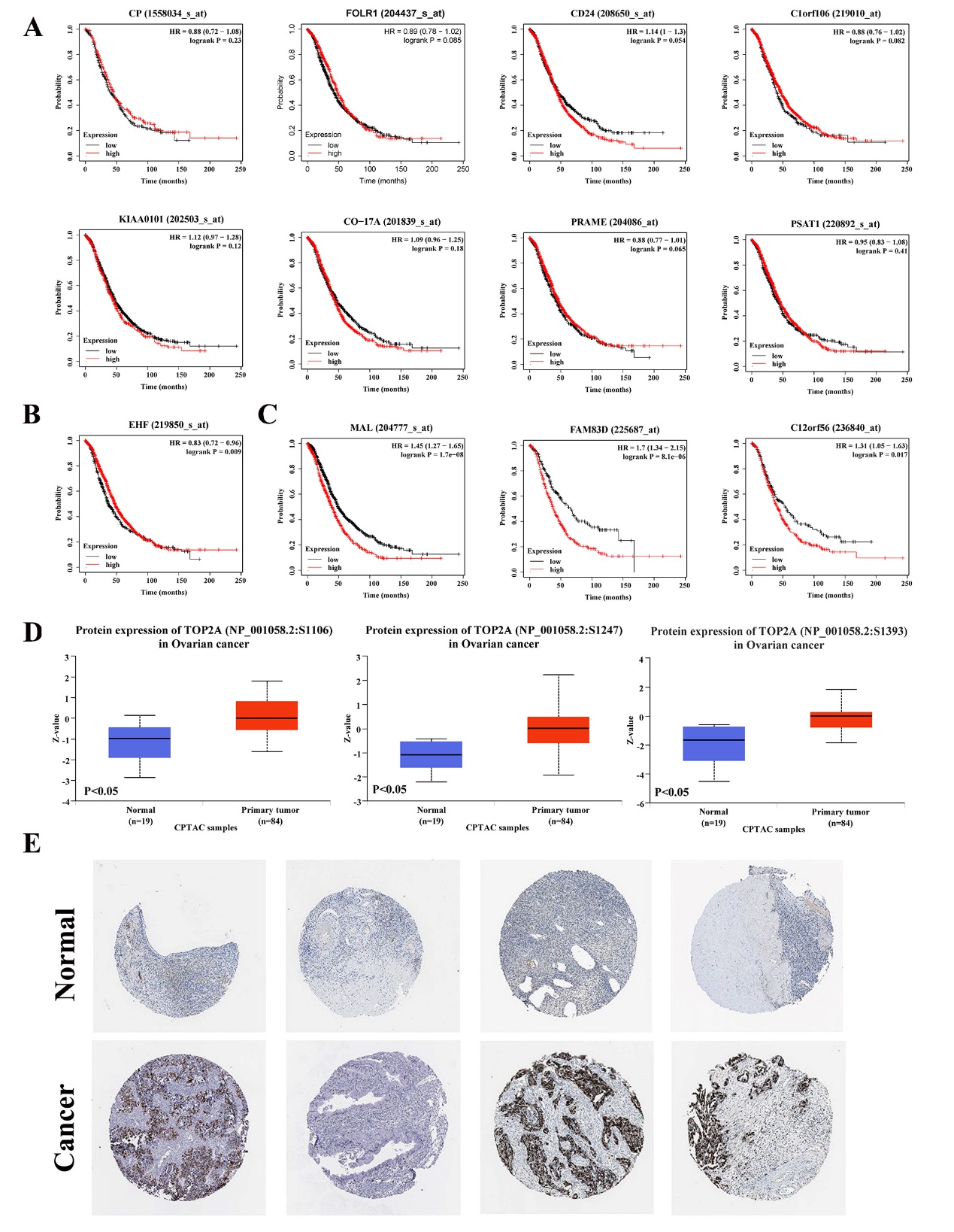
**Figure S2.** Prognostic significance of up-regulated genes in OC and IHC results of TOP2A protein. (A) CP, FOLR1, CD24, C1orf106, KIAA0101, CO-17A, PRAME and PSAT1 gene had no significant correlations with OS of patients with ovarian cancer. (B) Low expression of EHF gene was linked to a poor OS of ovarian cancer. (C) High expression of MAL, FAM83D, C12orf56 gene was associated with a poor OS of ovarian cancer. (D) In three data sets, p-TOP2A protein expression was higher in ovarian cancer tissues than in normal tissues. (E) The IHC staining of TOP2A protein in normal and OC tissues.


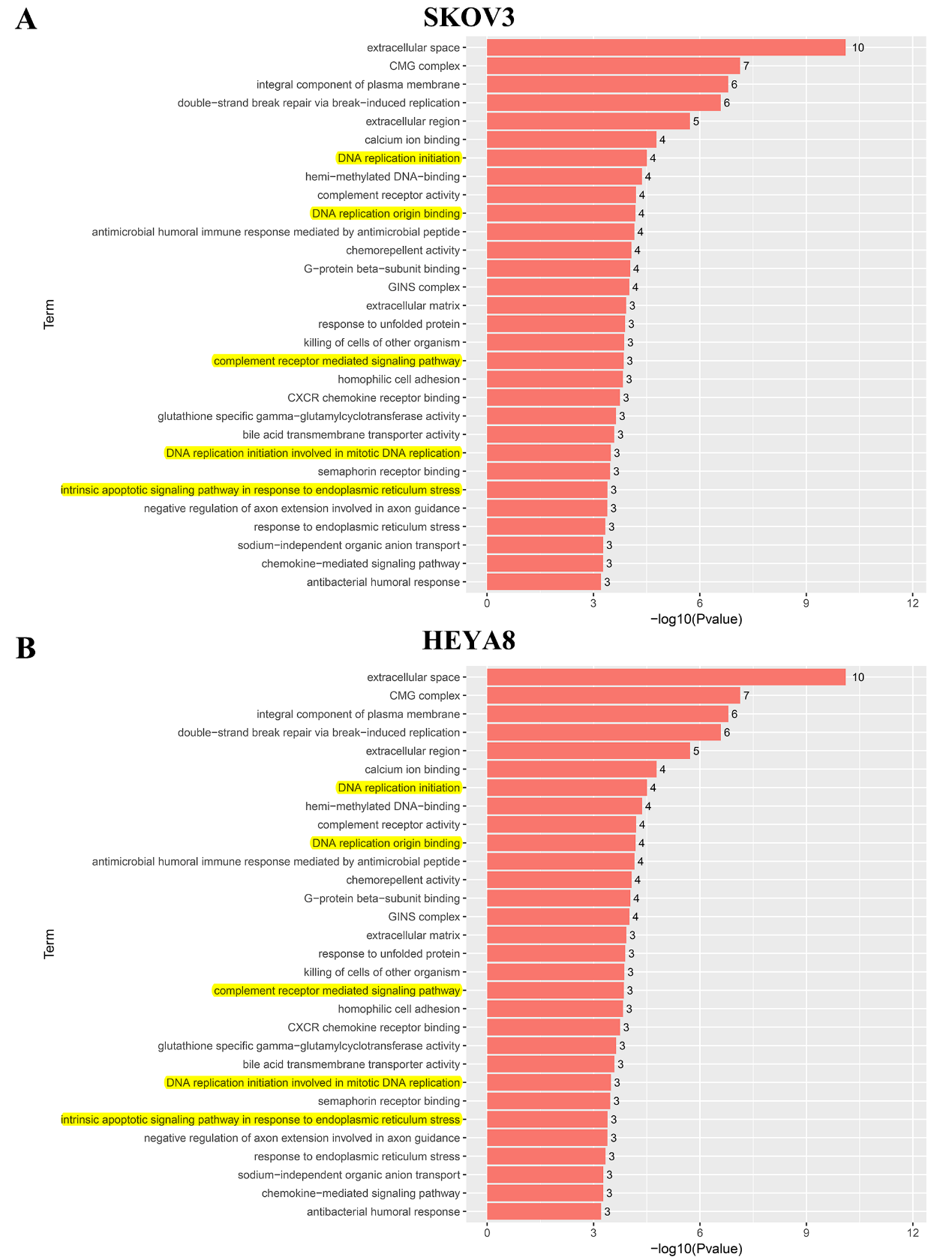
**Figure S3.** Results of GO enrichment analysis of RNA-seq. (A) GO enrichment analysis of SKOV3. (B) GO enrichment analysis of HEYA8.
